# The impact of extended electrodiagnostic studies in Ulnar Neuropathy at the elbow

**DOI:** 10.1186/1471-2377-9-52

**Published:** 2009-10-09

**Authors:** Kari Todnem, Ralf Peter Michler, Tony Eugen Wader, Morten Engstrøm, Trond Sand

**Affiliations:** 1 Department of Neurology and Clinical Neurophysiology, St. Olav's University Hospital, Trondheim, Norway; 2 Department of Neuroscience, Norwegian University of Science and Technology, Trondheim (NTNU), Norway

## Abstract

**Background:**

This study aimed to explore the value of extended motor nerve conduction studies in patients with ulnar nerve entrapment at the elbow (UNE) in order to find the most sensitive and least time-consuming method. We wanted to evaluate the utility of examining both the sensory branch from the fifth finger and the dorsal branch of the ulnar nerve. Further we intended to study the clinical symptoms and findings, and a possible correlation between the neurophysiological findings and pain.

**Methods:**

The study was prospective, and 127 UNE patients who were selected consecutively from the list of patients, had a clinical and electrodiagnostic examination. Data from the most symptomatic arm were analysed and compared to the department's reference limits. Student's t - test, chi-square tests and multiple regression models were used. Two-side p-values < 0.05 were considered as significant.

**Results:**

Ulnar paresthesias (96%) were more common than pain (60%). Reduced ulnar sensitivity (86%) and muscle strength (48%) were the most common clinical findings. Adding a third stimulation site in the elbow mid-sulcus for motor conduction velocity (MCV) to abductor digiti minimi (ADM) increased the electrodiagnostic sensitivity from 80% to 96%. Additional recording of ulnar MCV to the first dorsal interosseus muscle (FDI) increased the sensitivity from 96% to 98%. The ulnar fifth finger and dorsal branch sensory studies were abnormal in 39% and 30% of patients, respectively. Abnormal electromyography in FDI was found in 49% of the patients. Patients with and without pain had generally similar conduction velocity parameter means.

**Conclusion:**

We recommend three stimulation sites at the elbow for MCV to ADM. Recording from FDI is not routinely indicated. Sensory studies and electromyography do not contribute much to the sensitivity of the electrodiagnostic evaluation, but they are useful to document axonal degeneration. Most conduction parameters are unrelated to the presence of pain.

## Background

Ulnar nerve neuropathy at the elbow (UNE) was described in 1922 by Buzzard [1] who had had personal experience with UNE. In 1958 Feindel and Stratford [2] called the condition "cubital tunnel syndrome" in order to indicate that its cause was assumed to be a compression at the humeroulnar arcade. However, lesions at the ulnar groove account for most cases [3]. A standardized incidence rate of 20.9 cases per 100 000 persons per year has been found [4]. In 1956 Simpson [5] described a neurophysiological method for the diagnosis of UNE, and new methods have often been proposed and discussed. Extended protocols like inching, recording from multiple muscles, and conduction block evaluation have been evaluated [6-8], but there is no general agreement about the optimal diagnostic procedure.

The clinical diagnosis of UNE can be difficult, and pain may or may not be present. Gilliat and Thomas [9] stated that they never had seen abnormal ulnar nerve conduction in patients with complaints of pain or paresthesia but without physical signs on clinical examination. Eisen [10] on the other hand found reduced motor conduction velocity (MCV) across sulcus in 10 of 56 patients with clinically mild ulnar lesions. Indeed, the relationship between pain and electrophysiological findings in UNE has not been much studied.

The purpose of this study was to compare the sensitivity of our standard neurophysiological method for the diagnosis of UNE to an extended protocol. We specifically wanted to evaluate the use of a mid-sulcus stimulation site over the ulnar nerve at the elbow, and to compare recording from the abductor digiti minimi muscle (ADM) to recording from the first dorsal interosseus muscle (FDI) in order to find the most sensitive and least time-consuming effective method. Further we wanted to evaluate the utility of examining both the sensory branch from the fifth finger and the dorsal branch of the ulnar nerve. Finally, we intended to study if pain related to other clinical symptoms and findings could predict the neurophysiological severity of ulnar neuropathy. In the present study we reported that three stimulation sites in the elbow for MCV to ADM was the most effective method.

## Methods

### Patients

The study was prospective, and 127 UNE patients were selected consecutively from the list of patients examined in our department from 2002 - 2008. The clinical and electrodiagnostic examinations were performed once in each patient. The clinical inclusion criteria were based on the symptoms and neurological findings indicative of ulnar nerve entrapment [11]. The electrodiagnostic inclusion criterium was at least one abnormal neurophysiological findings in the ulnar nerve when a standard protocol was used, i.e. either reduced MCV across the ulnar groove (10 cm segment: < 50 m/s), reduced ADM amplitude (< 4.3 mV), or sensory conduction velocity (SCV) (< 48 m/s) and amplitude (< 2 μV), according to our department's reference values (n = 116 nerves from 100 subjects (15 healthy hospital employees and 85 patients controls without neurological signs or disease, i.e. discharged with a symptoms diagnosis, mean age 42 years, range 14 - 78 years, mean height 169 cm, range 154 - 198 cm). Subjects with normal neurophysiological results were not included in the study regardless of their symptoms and neurological findings. The extended neurophysiological examination protocol (see below) was unchanged for the study period. The nerve conduction examinations were first performed by a neurophysiology technician and then controlled (and rechecked if necessary) by one of the four attending physicians, who also performed the electromyography (EMG).

Exclusion criteria included either clinical or electrodiagnostic evidence of ulnar nerve entrapment at the wrist, cervical radiculopathy, polyneuropathy or significant suspicion of carpal tunnel syndrome (CTS) or another clinical condition apart from UNE.

The mean age of the patients was 50.5 (range 22 - 89; median 52) years, 42 (33%) women and 85 (67%) men. The mean height of the patients was 175 (154 - 193, SD = 8.8) cm. The duration of UNE symptoms at presentation varied from 1 - 576 (mean 24.2, median 7.8) months. Seventy-four patients (58%) had left ulnar nerve symptoms, 26 (21%) had ulnar symptoms in the right side and 27 (21%) had symptoms from both ulnar nerves. Fifty-six patients (44%, n = 125) had had no other diseases, 12 (9.4%) had had neck symptoms, 9 (7.1%) had diabetes, 6 (4.7%) had had lumbar spine symptoms, 5 (3.9%) had had CTS, 5 (3.9%) had had heart disease, 4 (3.1%) had increased blood pressure, 3 (2.4%) had had cancer and the rest had or had had a variety of different diseases. Five (3.9%) had experienced trauma to one elbow, 4 (3.1%) had had epicondylitis, one used elbow crutches and one had previously been operated for UNE.

### Neurophysiological examinations

Motor and orthodromic sensory conduction studies of the ulnar and median nerves were performed bilaterally. All studies were performed with the Dantec Keypoint apparatus (Dantec Medical A/S, Skovlunde, Denmark), using previously described methods [12,13]. The ulnar nerve was stimulated at the wrist, 4 cm below the midpoint of the ulnar sulcus in the elbow, in the sulcus, 6 cm above the sulcus midpoint and 10 cm proximally in the axilla, with surface recording electrodes over both the ADM and FDI. The sensory ulnar nerves were stimulated with ring electrodes at the 5th finger and the orthodromic sensory nerve action potential (SNAP) was recorded with surface electrodes at the wrist. The dorsal sensory ulnar branch was stimulated with a saddle electrode at the dorsum of the hand and recorded at the wrist. Skin temperature was measured and kept above 32°C [11] by means of heated packs and a heating lamp. During the examination of the ulnar nerve the arm was externally rotated at the shoulder and the elbow flexed 70-90° [11,14,15]. Reference values for the standard protocol, with 10 cm distance between stimulation points in the elbow, were our department's ordinary reference values (see above). Reference values for the smaller segments (6 cm above and 4 cm below the midpoint of the ulnar sulcus) were calculated from forty-five non-symptomatic arms within the study group (28 men, 17 women, age 42 years, range 22-67 years) with normal median and standard ulnar MCV and SCV. Only patients with no disease (n = 38), migraine (n = 1), neck pain (n = 4) and epicondylitis (n = 2), were accepted. EMG with a concentric needle electrode was performed in the FDI, and also in ADM and the abductor pollicis brevis (APB) if relevant in selected patients. EMG was categorized as "normal" or "neuropathic" according to our standard EMG reference values.

### Clinical neurological examinations

Paresthesias, numbness, pain in the elbow, forearm and hand, and the feeling of reduced muscle power were recorded. Sensitivity to pin-prick pain and light touch, presence of muscle atrophy and reduced muscle strength in the hands and arms were noted.

The research protocol was approved by the local institutional review board and is in compliance with the Helsinki Declaration. Patients received written information about our nerve conduction study procedure before they consented to come for the examination, and they were also informed about the procedure on the day of the examination by experienced technicians.

### Statistical analysis

Amplitudes were log-transformed before the reference limits were computed. In order to compensate for possible variation in the distribution between the parameters we used the mean of three limits (mean - 2 SD, minimum value (maximum value for latencies), and lower (upper for latencies) 2.5% percentile.

In UNE patients, data from the most symptomatic arm were analysed. In patients with bilateral symptoms and bilateral conduction abnormalities, the right side was chosen.

Conduction block was defined as at least 40% reduction in amplitude at site 3 (in sulcus), 4 (above sulcus) or 5 (below axilla) compared to site 1 (wrist). The upper amplitude reduction limit was 25% in the laboratory's reference limits and 33% in the present non-symptomatic side reference sample.

Patients were grouped according to no pain versus pain in the hand, forearm and elbow. CV parameters in subgroups were compared with Student's t - test. Frequencies were compared with chi-square tests. The relation between ulnar MCV and sensory amplitude (dependent variables) and age, sex, height and symptom duration was explored in multiple regression models. Two-side p-values < 0.05 were considered as significant. SYSTAT V.11 and SPSS v.15 statistical packages for Windows were used.

## Results

In all cases of incomplete data set for different variables the total number of examined patients are given.

### Clinical symptoms

Ulnar paresthesias occurred in 117 of 122 patients (96%) and subjectively reduced ulnar sensitivity occurred in 98 of 121 patients (81%). Altogether 72 of 120 patients (60%) had pain. Elbow pain (50 patients, 41%) was reported somewhat more frequently than hand (42 patients, 36%) and forearm (33 patients, 28%) pain. Sixty-four of 120 (53%) reported reduced muscle strength in the hand.

### Clinical neurological findings

are shown in Table 1.

**Table 1:** The clinical findings in 127 UNE patients (mean age 50.5 years, median 52, range 22 - 89)

**Clinical findings**	**Patients with clinical findings**	**Percent**	**Number of patients^1^**
Ulnar muscle atrophy	29	23.2	125

Median muscle atrophy	1	0.8	125

Radial muscle atrophy	0	0	125

Reduced ulnar muscle strength	59	47.6	124

Reduced median muscle strength	0	0	124

Reduced radial muscle strength	0	0	123

Reduced ulnar sensitivity in the hand	107	86.3	124

Other reduced sensitivity in the hand	6	4.7	124

Reduced D-M sens. in the forearm	16	12.9	124

D-M sens., reduced sensitivity distal and medial. The reduced ulnar muscle strength was weakness in finger spreading, abduction of the fifth finger and flexion of the fourth and fifth fingers. ^1^The numbers are less than 127 because information was missing in 2 - 4 patients.

### Neurophysiological findings

The MCV parameters for ADM and FDI in the most symptomatic arm in all patients are displayed in Table 2. The table show that with two stimulation points the across sulcus segment were abnormal in 80% of the patients which give 80% sensitivity for the old protocol. The sensitivities for MCV at the different elbow segments are very generally similar for ADM and FDI. For the corresponding amplitudes the sensitivities were generally higher for FDI.

**Table 2:** The ulnar motor nerve parameters in 127 UNE patients (most symptomatic arm)

**Motor parameters**	**Abductor digiti minimi muscle**			**First dorsal interosseus**		
	**Mean (sd)**	**Range**	**% abnormal (ref.lim)**	**Mean (sd)**	**Range**	**% abnormal (ref.lim)**

Distal latency (ms)	3.0 (0.4)	(2.2-4.6)	7% (3.6)	3.6 (0.5)	(2.3-5.3)	6% (4.4)

Forearm MCV (m/s)	56 (6)	(36-71)	21% (52)	55 (6)	(32-70)	17% (51)

Below sulcus (m/s)	45 (16)	(13-67)	54% (49)	45 (14)	(9-67)	40% (44)

Above sulcus (m/s)	44 (14)	(10-70)	69% (48)	42 (14)	(10-75)	62% (45)

Across sulcus (m/s)	41 (9)	(11-67)	80% (49)	40 (8)	(10-59)	84% (48)

Upper arm (m/s)	62 (7)*	(35-77)	6% (51)	62 (7)†	(33-77)	7% (51)

F-M (ms)	26.1 (2.3)	(19.0-33.6)	31% (27.3)			

						

Wrist amplitude (mV)	8.2 (2.4)	(1.0-14.0)	16% (6.3)	8.7 (3.8)	(0.2-18.2)	33% (7.3)

Below sulcus (mV)	7.3 (2.5)	(1.0-13.1)	20% (5.1)	7.3 (3.7)	(0.1-17.3)	28% (5.1)

In sulcus (mV)	6.8 (2.6)	(0.9-12.9)	25% (5.3)	6.7 (3.5)	(0.1-17.1)	33% (5.2)

Above sulcus (mV)	6.0 (2.6)	(0.4-12.7)	35% (5.2)	5.8 (3.4)	(0.1-14.7)	44% (5.1)

Upper arm (mV)	5.9 (2.6)‡	(0.4-11.7)	34% (5.0)	5.7 (3.4)	(0.0-14.4)	56% (6.0)

						

Relative amplitude	0.70 (0.24)	(0.07-1.36)	30% (0.67)	0.64 (0.27)	(0.00-1.10)	34% (0.58)

*Co-stimulation of the ulnar and the median nerve in 5 patients and not used; †Co-stimulation of the ulnar

and the median nerve in 13 patients and not used; ‡Not recorded in one patient.

Recording from ADM we found reduced MCV in 68 arms below sulcus ulnaris (4 cm segment, mean MCV = 33 m/s) and in 87 arms above sulcus (6 cm segment, mean MCV = 36 m/s). Thirty-three subjects had abnormal MCV both above and below sulcus. From these numbers can be calculated that 96% of the patients had abnormal MCV with the new protocol with three stimulation points in the elbow, which give a sensitivity of 96%. The sensitivity increased with 16% by adding a third stimulation point in the elbow. MCV across the elbow (over both segments: 10 cm) was abnormal in 102 of 127 subjects with mean MCV = 38 m/s. Twenty patients with normal MCV across the elbow had abnormal fractionated MCV either below or above the ulnar groove. We found significant conduction block (amplitude decrement > 40% at or above the elbow) in 33 patients. Sixty-five percent of those with block had ulnar motor weakness and/or atrophy on clinical examination compared to 40% of those without block (p = 0.005, chi-square test). Blocking was not related to symptom duration (mean 23.3 month versus 25 months, p = 0.9). Only two nerves with normal across-sulcus MCV to ADM had block, but both had abnormal MCV either below or above sulcus. Forty-four (35%) patients had electrodiagnostic entrapment of the ulnar nerve at ADM in both elbows.

With recording electrodes over the FDI muscle, we found MCV abnormalities below, above and across sulcus in 51, 79 and 107 subjects (only 17 nerves were abnormal both over and below sulcus). Only three subjects (2%) with normal MVC over ADM across, below and above sulcus had abnormal MCV recorded over FDI. Five subjects (4%) with abnormal MCV over ADM across, below and above sulcus had normal MCV recorded over FDI.

The results from the ulnar sensory nerve parameters are seen in Table 3.

**Table 3:** The ulnar sensory nerve parameters in 127 UNE patients (most symptomatic side)

**Sensory parameters**	**Mean (sd)**	**Range**	**% abnormal (ref.lim)**
Finger 5 amplitude (uV)	3.3 (2.8)	(0.0-13.7)	35% (1.7)

SCV finger 5 (m/s)	53.9 (6.4)*	(37.0-71.0)	17% (47.3)

Dorsal branch amplitude (uV)	5.0 (4.7)†	(0.0-23.0)	27% (2.0)

SCV dorsal branch (m/s)	57 (8)‡	(24-74)	6% (46.6)

*The potential was unelicitable (0 μV) in seven patients; †Not recorded in three patients; ‡The potential was unelicitable (0 μV) in 18 patients

The amplitude of the sensory nerve from the fifth finger was absent in 13, reduced in 31 and normal in 83 (65%) patients. The dorsal ulnar nerve action potential was absent in 18, reduced in 15 and normal in 91 (73%) patients. Either amplitude or SCV was abnormal in 39% and 30% respectively. In eleven patients with normal sensory findings in the fifth finger we found abnormal dorsal branch SCV or amplitude abnormality. In only one patient with normal MCV over, under and across sulcus (ADM) we found abnormal dorsal ulnar sensory amplitude.

The results from the median nerves were used only for differential diagnosis. All together 29 patients had slightly abnormal distal motor velocity (APB) or SCV (third finger or vola) on one or both sides compatible with non-symptomatic CTS.

Patients with and without pain had generally similar CV parameter means. F-responses were slightly longer (26.7 versus 25.7, Student's t-test, p = 0.02) and MCV was decreased above sulcus in those without pain (40.2 versus 45.9 m/s, p = 0.028). Pain was not related to abnormality rates for motor (ADM or FDI) or sensory (fifth finger or dorsal branch) ulnar conduction, apart from less conduction block in those with pain (32%) compared to those without pain (50%, p = 0.047, chi-square test). Pain was not correlated with paresthesia, reduced sensitivity, reduced ulnar strength or ulnar muscle atrophy (chi.-square p > 0.18).

EMG was recorded from FDI in 124 patients (49% abnormal). Abnormal EMG in FDI was found in 54% of 24 patients with abnormal standard MCV across the elbow, and in 29% of 100 patients with normal MCV (chi-square p = 0.03). Abnormal EMG in FDI was found in 52% of those with pain and in 42% of patients without pain, a non-significant difference (chi-square test, p = 0.26). EMG was recorded from ADM in 40 patients (53% abnormal), and from APB in only 23 patients with no abnormal findings.

## Discussion

### Electrodiagnosis

The results from the electrodiagnostic (ED) studies showed that 16% of patients with electroclinical UNE were not diagnosed by the use of only two stimulation sites below and above the ulnar groove. Consequently, we will advocate three stimulation sites in the elbow. Smaller distances in segmental nerve conduction studies are probably safe with modern electrodiagnostic equipment [16]. Recording from ADM and FDI gave very much the same results, as shown in Table 2[17-19]. Conduction velocity in the forearm while recording from ADM was abnormal in 21% of the patients, most probably caused by pronounced axonal degeneration in the nerve. We found a significant correlation between no pain in the elbow and decreased MCV above sulcus, which may indicate that entrapment in the ulnar groove is less painful than in the cubital tunnel. Blocking of thin myelinated A-delta fibres can explain this association. A general lack of correlation between pain and ED is not unexpected as thin-fibres function is not reflected in CV parameters. However, we could not confirm that patients with pain should be less likely to show abnormal CV for the majority of parameters.

Fibres to FDI may be more susceptible to demyelination compared to fibres to ADM [20]. However, only 2.3% of the patients had normal results in the ADM recording and abnormal findings in the FDI recording. The most cost-effective procedure is consequently to use only ADM recordings, with optional recording over FDI, and additional use of the inching technique [6,8] in selected cases. The advantages of the new protocol are that it can be performed as a screening test by the neurophysiology technicians. There is less risk of electrical spreading due to longer distances between the nerve stimulation points (six and four cm), while the segment steps in the inching technique are one or two cm. However, this study did not aim to compare the new protocol and the well known inching technique. Inching is also a superior technique in experienced hands for precise localization of ulnar nerve entrapment at the elbow.

Significant conduction block (amplitude decrement > 40%) at the elbow were found in 26% of the patients. This is a definite sign of nerve entrapment, but it was a rather infrequent finding in this UNE study, and it was not related to symptom duration. In another study [21] of 244 UNE patients 16% had motor conduction block (amplitude decrement ≥ 50).

The sensory nerve from the fifth finger was abnormal in 39% of the patients, and 30% had an abnormal dorsal ulnar nerve study. These results are in contrast to the large prevalence of sensory symptoms as 96% reported ulnar paresthesias and 86% had objectively reduced ulnar sensitivity in the hands. This discrepancy can be explained by a selection bias because patients with more severe symptoms and signs tend to be admitted for electrodiagnostic studies. The ulnar sensory findings in UNE patients in other studies vary from zero abnormal distal findings [6] to 51 - 55% abnormal sensory studies across the elbow [7,22]. However, even if sensory studies do not contribute much to the sensitivity of the electrodiagnostic evaluation, they are useful to document axonal degeneration or severe dispersion within sensory fascicles.

We found abnormal EMG in about 50 percent of the UNE patients and abnormal MCV (as opposed to conduction block) was moderately associated with an increased rate of neuropathic EMG. Bhala [23] found abnormal EMG in 78 percent of patients with reduced NCV < 45 m/s) across the elbow with the highest abnormality rate in FDI. However, in our study only 31% of the UNE patients had EMG from ADM (53% abnormal), while 98% had EMG from FDI (49% abnormal). A more balanced examination rate might have changed these figures.

### Clinical findings and previous diseases

Ninety-six percent of the UNE patients reported paresthesias in the ulnar territory, 86% had objectively reduced ulnar sensitivity in the hand, 48% had reduced ulnar muscle strength and 41% had pain in the elbow. Similar findings occurred among patients who had 290 surgical procedures for UNE [24]. In only 6% of our patients we found reduced skin sensitivity outside the ulnar territory in the hand and 16% reported reduced sensitivity distal/medial in the forearm, which may be caused by a different anatomical nerve distribution or an unspecific pain-related dysfunction, as there were no clinical or electrodiagnostic evidence for another diagnosis in these patients. According to the inclusion criteria only patients with abnormal neurophysiological values of the ulnar nerve were included. Suspected UNE patients with normal electrodiagnostic values were consequently not included, and the symptom distribution and neurological findings might have been different with other inclusion criteria.

The frequency of previous CTS (3.9%) [25] and diabetes mellitus (7.1%) [26,27] were close to prevalences within the general population. Unexpectedly, only 4% had had trauma to the elbow. Ten percent had had neck symtoms, which is very common in the Norwegian society. Consequently, UNE in this study seemed to be a singular condition probably caused by injury of the nerve, statical flexed position of the elbow, simple overuse of one arm, genetic predisposition, or an inflammation or another local disease in the elbow region [1].

We found 79% subjectively affected left ulnar nerves and only 21% affected right nerves. Left ulnar nerve dominance in UNE is also found in other studies [19]. The right hand is dominant in most people and logically one would have expected the opposite, if ordinary work played a major role in the pathogenesis, which is found in CTS [28]. In this study UNE prevailed in men (67%), in concert with other studies [4,19,24,29]. Matev [30] reported that the ulnar nerve in men is more mobile and therefore more sensitive to gliding impairment at the medial epicondyle.

### The use of electrodiagnosis in UNE

Differential diagnosis involves many diseases concerning the spinal cord, cervical roots and other peripheral entrapment sites. Consequently, clinical findings and tests in our opinion are not sufficient to make a qualified diagnosis of UNE. In the literature most surgeons advocate electrodiagnosis for UNE [24], but others prefer clinical testing without electrodiagnosis [31]. Especially before surgery one should produce direct evidence for entrapment of the ulnar nerve at the elbow and single out other possible etiologies. Electrodiagnosis before surgery is also recommended in order to have a valid baseline for further studies on patients with residual symptoms after surgery. Patients might be in doubt whether to have an operation or not, and results from a nerve conduction study can help him/her to decide. Nonoperative management can also be successful, especially in patients with symptoms only [32].

There is no useful clinical gold standard for the diagnosis of UNE. Attempts to define gold standards based on operative success will also fail because peroperative complications, placebo effects and spontaneous remissions will interfere. Appropriately performed nerve conduction methods, used for more than 50 years, have proved to be very reliable, and most experts consider a combination of clinical and electroneurographic signs as a "gold standard" for the most common entrapments. Accordingly, we chose to apply electroclinical diagnostic inclusion criteria in the present study. The advantage is that the diagnostic precision is optimized, as it is recommended for the diagnosis of UNE [11]. We could accordingly compare sensitivities among the various extended parameters. However, it should be noted that the comparison between standard and extended parameters in the present study could have been biased in favor of the standard parameters because they were among the inclusion criteria.

In addition we did not study other groups and could accordingly not estimate specificities of the extended electrodiagnostic parameters. Inclusion of patients with a clinical picture suggestive of UNE without neurophysiological evidence of entrapment (using the standard protocol) would have enabled us to also estimate specificities. However, it should be noted that interpretation of specificity is somewhat ambiguous when a definite gold-standard does not exist. Indeed, it will be difficult to differ between true and false positives in a population with symptoms suggesting UNE. Accordingly, specificity should also be calculated in a healthy control group and preferably, in a group with different symptomatology, for instance carpal tunnel syndrome. The lack of such control groups is a weakness of the present study. Accordingly, we recommend to perform such controlled studies, as well as prospective studies, in order to estimate specificities for our extended electrodiagnostic parameters in different groups, as well as the prognostic value of the extended parameters.

## Conclusion

Three stimulation sites in the elbow for MCV to ADM seems to be a useful and sufficiently sensitive method in the diagnosis of UNE, while recording from FDI is not routinely indicated. Sensory studies and electromyography do not contribute much to the sensitivity of the electrodiagnostic evaluation, but they are useful to document axonal degeneration. Most conduction parameters are unrelated to the presence of pain. We advocate the use of a neurophysiological examination in the diagnosis of UNE to single out other differential diagnoses, because symptoms and findings in this condition are not uniform.

## Abbreviations

ADM: abductor digiti minimi muscle; APB: abductor pollicis brevis muscle; CTS: carpal tunnel syndrome; CV: conduction velocity; ED: electrodiagnosis; EMG: electromyography; FDI: first dorsal interosseus muscle; MCV: motor nerve conduction velocity; SCV: sensory nerve conduction velocity; UNE: ulnar nerve entrapment at the elbow.

## Competing interests

The authors declare that they have no competing interests.

## Authors' contributions

KT and RPM designed the study and KT wrote the primary manuscript. KT, RPM, TEW and ME collected the data. TS performed the statistical analysis, participated in the interpretation of data and revised the manuscript critically. All authors read and approved of the final manuscript.

## Authors' information

KT, RPM, TEW and TS are specialists in clinical neurophysiology and neurology, ME is specializing in clinical neurophysiology, and all work with peripheral nerve entrapment problems. KT has a PhD grade. TS is a professor in clinical neurophysiology.

## Pre-publication history

The pre-publication history for this paper can be accessed here:


